# Aspiration-mediated hydrogel micropatterning using rail-based open microfluidic devices for high-throughput 3D cell culture

**DOI:** 10.1038/s41598-021-99387-6

**Published:** 2021-10-07

**Authors:** Dohyun Park, Jungseub Lee, Younggyun Lee, Kyungmin Son, Jin Woo Choi, William J. Jeang, Hyeri Choi, Yunchan Hwang, Ho-Young Kim, Noo Li Jeon

**Affiliations:** 1 Department of Mechanical Engineering, Seoul National University, Seoul, 08826 Republic of Korea; 2 Department of Materials Science and Engineering, Northwestern University, Evanston, IL 60208 USA; 3 Interdisciplinary Program for Bioengineering, Seoul National University, Seoul, 08826 Republic of Korea; 4 Department of Electrical Engineering and Computer Science, Seoul National University, Seoul, 08826 Republic of Korea; 5 Bio-MAX Institute, Seoul National University, Seoul, 08826 Republic of Korea

**Keywords:** Biotechnology, Engineering

## Abstract

Microfluidics offers promising methods for aligning cells in physiologically relevant configurations to recapitulate human organ functionality. Specifically, microstructures within microfluidic devices facilitate 3D cell culture by guiding hydrogel precursors containing cells. Conventional approaches utilize capillary forces of hydrogel precursors to guide fluid flow into desired areas of high wettability. These methods, however, require complicated fabrication processes and subtle loading protocols, thus limiting device throughput and experimental yield. Here, we present a swift and robust hydrogel patterning technique for 3D cell culture, where preloaded hydrogel solution in a microfluidic device is aspirated while only leaving a portion of the solution in desired channels. The device is designed such that differing critical capillary pressure conditions are established over the interfaces of the loaded hydrogel solution, which leads to controlled removal of the solution during aspiration. A proposed theoretical model of capillary pressure conditions provides physical insights to inform generalized design rules for device structures. We demonstrate formation of multiple, discontinuous hollow channels with a single aspiration. Then we test vasculogenic capacity of various cell types using a microfluidic device obtained by our technique to illustrate its capabilities as a viable micro-manufacturing scheme for high-throughput cellular co-culture.

## Introduction

Proper understanding of interactions between various cell types is essential in tissue engineering and design of in vitro organ models^1,2^. Microfluidic co-culture platforms embedding multiple cell types suspended in hydrogels can recapitulate specific organ functions and thus provide insights into complex physiological systems^3,4^. Microstructures within these platforms facilitate the arrangement of cells into physiologically relevant layouts^5^. For example, micropillars^6^ or bumps (named as "phageguide")^7^ constructed on hydrophobic surfaces form narrow gaps between microchannels, resulting in a high bursting pressure for a liquid to infiltrate into adjacent microchannels. When a hydrogel precursor is loaded into a microchannel with lower pressure than the bursting pressure of the gap, the liquid will completely fill the microchannel without disturbing adjacent channels. Different types of cell suspensions or hydrogels containing cells can later fill the remaining adjacent channels after cross-linking of the pre-loaded hydrogel precursor. These microstructure-mediated hydrogel patterning methods can mediate meniscus-pinning for precisely segregating co-cultures in hydrogels to model vasculature^8–10^, tumor extravasation dynamics^11^, glomerular filtration barrier^12^, and intestinal epithelium tubes^13^. The hydrogel loading guided by surrounding microstructures, however, fails when the hydrogel pressure exceeds a limit, or capillary-bursting pressure, causing the gel to invade the undesired region^14,15^. Thus, a great care should be taken to limit the injection pressure, resulting in yield variation depending on the operator’s pipetting skill and low throughput associated with short available time of hydrogel precursor^16^. Furthermore, the complex microscale features of such devices, achieved typically through soft lithography, preclude application of scalable manufacturing processes like injection molding.

Open microfluidic devices that utilize spontaneous capillary wicking along rail structures present an alternative avenue for gel patterning without relying on the capillary-burst valve^17–22^. Here, rails denote long hydrophilic plates between which liquids are confined owing to capillary forces, while the side areas are exposed to the air^23^. In those studies, spontaneous capillary flow along corner or rail structures enhanced yield by reducing the effect of user’s dispensing pressure. Furthermore, their structures are simple to produce with injection molding so that the open microfluidic design also enhanced productivity. However, the air–liquid interfaces in rail-based microfluidics are highly sensitive to the volume of liquid dispensed, thus requiring a careful loading process with accurate volume of cell-laden hydrogel precursors^24^ or requiring additional design for rail structures to reduce the reliance on a precise volume^17,25^.

In our previous study, we have introduced an aspiration-mediated hydrogel micropatterning technique within a rail-based open microfluidic device and utilized it for 3D immune cell cytotoxicity assay^26^. Even though some aspiration-mediated patterning within microfluidic systems have been introduced^18,27–29^, they were limited to provide full understanding of the patterning mechanism under rail structures. Here, we further characterize the aspiration-mediated patterning method under unique rail structures which yields agile, easy and accurate positioning of hydrogels with only a simple pipetting activity. The scheme relies on the capillary holding capacity of rail-based microstructures against aspiration upon their geometry, which can be tailored to leave hydrogel precisely in desired location. Although delineated below in detail, the working principle is based on the dynamic characteristics of a solid–liquid-gas contact line that it starts to recede when the local contact angle gets smaller than the critical receding angle. By defining where the recession starts and stop with geometric parameters like aspiration port diameter and rail height, we can selectively remove hydrogel from undesired regions. We characterized design rules for successful patterning based on theoretical analysis and experimental verification using simplified structures. Proper design provides control over the sequence of microchannel formation, which enables the formation of multiple discrete channels with a single aspiration. A proof-of-concept device featuring four microchannels separated by hydrogel structures is shown to be able to co-culture five distinct cell types. Our rail-based open microfluidic devices are amenable to fabrication by injection molding^21,26^, thereby suggesting their great potential as an easy-to-use co-culture platform with low cost and high manufacturing scalablity.

## Results and discussion

**Figure 1:**
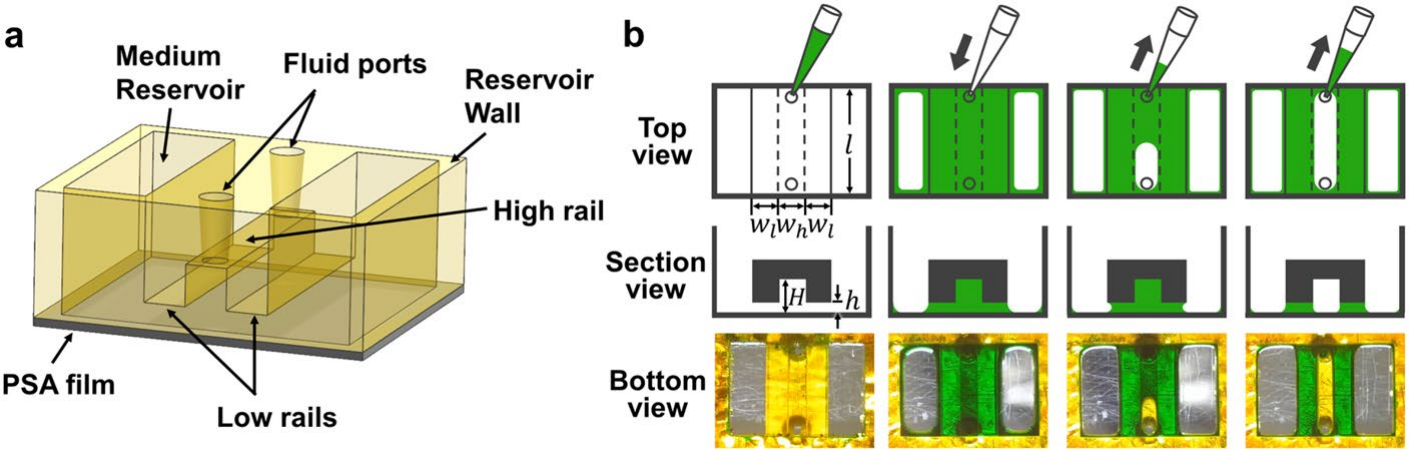
Schematic diagram of aspiration-mediated patterning within an open microfluidic device. (**a**) Schematic of a rail-based open microfluidic device, which is designed to yield a single channel beneath the high rail. (**b**) Illustration of steps in the aspiration-mediated microchannel formation process. The bottom row features optical images taken through the clear PSA film from the underside of the device during filling and aspiration of green dyed water.

Our rail-based open microfluidic platform consists of a 3D printed structure of photo curable resin and an underlying pressure sensitive adhesive (PSA) film. 3D-printed rail structures consisted of high rails (HRs) surrounded by low rail (LRs) and reservoir walls which supports rail structures and are bonded to the PSA film. HRs and LRs have different heights from the PSA film to retain liquid with different strengths (Fig. 1a). Figure 1b illustrates the basic working principle of the novel patterning process of hydrogel, which allows us to form hollow microchannels surrounded by precisely defined hydrogel patterns. The length of the rail, *l*, is 5 mm, and the width of low and high rail, ${w}_{l}$ and ${w}_{h}$, is uniformly 1 mm. The heights of low and high rails are respectively *h* = 100 μm and *H* = 300 μm. Two through-holes at the ends of a high rail function as fluid ports for liquid injection and aspiration. Air plasma treatment activates the surface of the device by increasing hydrophilicity. We first inject a hydrogel precursor solution through one of the fluid ports, which fills the area under all rails (the second column of Fig. 1b). Upon aspirating through the same port, the liquid is sucked into the pipette only from the region below high rail (HR), while the low rail (LR) strongly retains the liquid (the third column of Fig. 1b). The result is the hollow microchannel defined by the empty region under the high rail with the hydrogel precursor solution remaining pinned by the low rail (the fourth column of Fig. 1b). In the following, we delineate the physical principle behind this selective removal and controlled pinning of hydrogel patterns, which leads to various hydrogel-based channel structures and facile fabrication of cell co-culture systems.

### Theoretical analysis of microchannel formation

**Figure 2:**
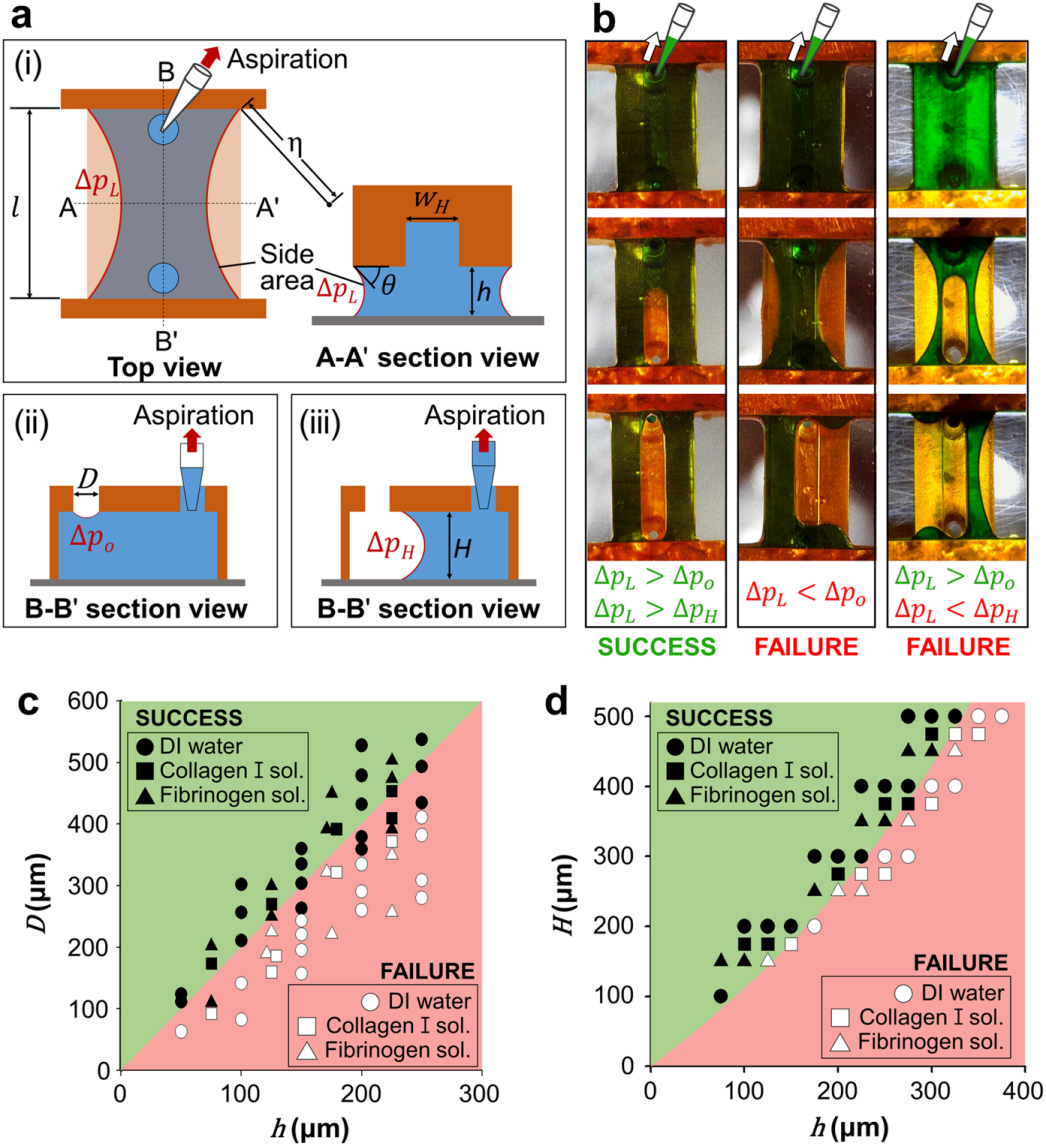
Theoretical considerations for designing rail structures capable of aspiration-mediated liquid patterning. (**a**) Schematic diagrams indicating key device dimensions and the Laplace pressures of menisci (**i**) immediately after aspiration, (**ii**) as the port interface deforms, and (**iii**) as the receding interface travels along the HR. (**b**) Time-lapse images from top to bottom illustrating meniscus dynamics during aspiration under three different combinations of critical capillary pressures. The dimensions of the structures are such that [*H*, *h*, *D*] = [300, 100, 300] μm, (Left column), [300, 200, 300] μm (middle column), [200, 175, 500] μm (Right column). Lengths and widths of all rails are fixed as 5 and 1 mm, respectively. (**c**) Plots of the effects of port diameter and LR height on bursting at the open port. (**d**) Plots of the effects of HR height and LR height on formation of microchannel. (**c**,**d**) Shaded regions demarcate dimensions suitable (green) and unsuitable (red) for microchannel formation based on the theoretical calculations. Closed markers and open markers indicate successful and unsuccessful microchannel formation in experiments, respectively. Deionized water (filled circle), 2.5 mg/ml of fibrinogen solution (filled traingle), and 3 mg/ml of collagen type I solution (filled square) were used for experimental verification. Experimental results were derived through three consecutive successes or failures for each condition.

As the pressure at a port is lowered by aspiration with a micropipette, the interior pressure of the hydrogel precursor solution is also lowered. The Young–Laplace equation states that the liquid–gas interface of the hydrogel precursor solution originally filling the gaps caves inward (Fig. 2a(i—iii)) due to the pressure difference between the atmosphere (p_*a*_) and the liquid (p_*l*_): ∆p = p_*a*_ − p_*l*_ = γ(1/R_1_ + 1/R_2_), where γ is the gas–liquid interfacial tension and R_1_ and R_2_ are two radii of curvature in orthogonal planes intersecting the interface^30^. The contact line where the gas–liquid interface meets the solid surface is initially immobile while resisting the pulling force, because it can recede only when the contact angle is reduced below the critical receding contact angle, *θ*_R,c_. The patterning of hydrogel that originally fills the gaps under both HRs and LRs critically depends on which interface starts to recede first by reaching *θ*_R,c._

For the initial hydrogel solution-loaded configuration, liquid–gas interfaces arise at the remaining port opposite to the port of aspiration, and lateral faces of the fluid filling the gaps below the LRs. We will refer to the opposing port as an “open port” in the following analysis. For the open port of diameter *D*, the maximum pressure difference that the interface can withstand before the contact angle on the inner wall of HRs reaches zero, ∆p_O,_ corresponds to the pressure difference for a hemispherical interface with the maximum curvature of 4/D: ∆p_O_ = 4γ/D (Fig. 2a(ii)). The capillary pressure at the air–liquid interface beneath LRs is given by ∆p_L_ = γ(2cos*θ*/*h* + 1/*η*), where *θ* is the instantaneous contact angle of liquid, *h* is the height of the rail, and *η* is the radius of curvature on a plane parallel to the substrate. During aspiration, *θ* decreases until reaching *θ*_*R,c*_ when the capillary pressure is maximized. We experimentally found that *θ*_R,c_ is nearly zero for hydrogel precursor solution on the hydrophilized 3D printed part of resin and PSA film surfaces. Hence, we estimate cos*θ*_*R,c*_ to be 1 due to the small receding contact angle, *θ*_*R,c*_. Furthermore, *η* scales with *l*, the length of rails, and *h* <  < *l* for most rail designs, thus the 1/*η* term is negligible. Hence, we estimate the maximum pressure difference for the side area below LRs as ∆p_L_ = 2γ/*h.*

If ∆p_*O*_ < ∆p_*L*_, the critical liquid pressure for interface recession is reached at the open port before the side area. Once burst at the open port, the interface spontaneously bulges accompanying the decrease of interface curvature to meet the bottom substrate. Then the interface curvature is newly defined by the height (*H*) and width (*w*_*H*_) of HR (Fig. 2a(iii)). The critical capillary pressure that can induce interface recession beneath HRs is given by ∆p_*H*_ = 2γ(1/w_H_ + 1/H). If ∆p_*L*_ > ∆p_*O*_ > ∆p_*H*_, the liquid pressure is already low enough to ensure continuous recession of the liquid beneath HR. If ∆p_*L*_ > ∆p_*H*_ > ∆p_*O*_, further decrease of the liquid pressure following bursting of the open port can cause the liquid to recede along a lane beneath the HR. In short, both cases of ∆p_*L*_ > ∆p_*O*_ > ∆p_*H*_ and ∆p_*L*_ > ∆p_*H*_ > ∆p_*O*_ result in successful patterning. However, if ∆p_*H*_ > ∆p_*L,*_ the liquid will stop upon bursting at the open port and recede beneath LRs, rather than HRs, resulting in patterning failure.

Figure 2b displays the experimental results of hydrogel patterning for different geometries of microdevices. In the left column, ∆p_*L*_ is greater than both ∆p_*O*_ and ∆p_*H*_, and thus the interface recession occurs from the open port, continues beneath HR, to result in a straight empty lane flanked by the hydrogel beneath LR. In the middle column, the side areas beneath LRs burst before the open port because ∆p_*L*_ < ∆p_*O*_. In the right column, the open port bursts first because ∆p_*L*_ > ∆p_*O*_, but further decrease of the liquid pressure bursts the side areas beneath LRs before the interface from the open port starts to recede beneath HR.

Figure 2c,d display the experimental results of microchannel formation as a function of the geometry of device, the port diameter and the heights of low and high rails, while fixing the length and width of all rails to 5 mm and 1 mm, respectively. We tested three types of liquids: deionized water, 2.5 mg/ml of fibrin gel pre-solution, and 3 mg/ml of collagen type I pre-solution. After fully filling the area under a rail structure, we immediately aspirated the liquid with a manual micropipette by gradually increasing the suction pressure until one of the interfaces starts receding. In Fig. 2c, we can predict where the interface recession onsets upon aspiration, whose criterion is given by ∆p_*L*_ = ∆p_*O*_, or *D* = 2* h*. For D > 2* h*, we get ∆p_*L*_ > ∆p_*O*_, and thus the port bursts first, leading to successful channel formation in the beginning. Otherwise, the interface moves at the side area of LR before at the port, preventing the formation of the microchannel under HR. Figure 2d gives the condition of the height of HR to ensure that the interface formed from the open port keeps receding while the interface beneath LR remains pinned. The corresponding boundary is given by ∆p_*L*_ = ∆p_*H*_, or *H* = *h*/(1 − *h*). For *H* > *h*/(1 − *h*), we get ∆p_*L*_ > ∆p_*H*_, leading to continuous recession of the interface under the HR from the open port. Otherwise, the interface under LR starts to recede, eventually merging with the interface originated from the open port. We see in Fig. 2c,d that our theoretical predictions agree well with the experimental results regardless of the liquid type. As all interfaces formed by a liquid under a rail structure share the same interfacial tension, γ, the moving interface is determined only by the geometry of the rail structure. The physical properties of the liquids with surface tension and viscosity were measured with Smartdrop (FEMTOBIOMED.inc, Korea) and AR-G2 (TA instruments, USA) respectively as listed in Table 1.

**Table 1:** Physical properties of liquids used in the experiments.

Liquid	Surface tension (mN/m)	Viscosity (mPa s)
Water (at 25 °C)	72.9	0.89
2.5 mg/ml of fibrinogen solution (at 25 °C)	57.0	11.6
3 mg/ml of collagen type I (at 4 °C)	61.7	59.1

Viscosities of hydrogel pre-solutions are measured at a shear rate of 10/s.

To determine the effect of aspiration pressure on patterning, we conducted patterning tests by aspirating deionized water that filled the region under a 3D-printed basic rail structure whose D, h, and H are 600 μm, 100 μm, and 300 μm, respectively, with various aspirating pressures. For the designed parameters, maximum capillary pressures at each interface are ∆p_*L*_ = 1439 Pa, ∆p_*O*_ = 479.8 Pa, and ∆p_*H*_ = 311.9 Pa. To apply different aspiration pressures, we applied maximum aspiration pressures of four different types of micropipettes whose maximum handling volumes are 10, 20, 100, and 200 μl, respectively. Maximum aspiration pressures of the four micropipettes were measured by tracking the velocity of receding fluid interfaces in straight microfluidic channels as detailed in the supplementary information and Table S1. When we aspirated the water with 10 μl and 20 μl pipettes, whose maximum aspiration pressures are below 2000 Pa, the water remained only under LRs as predicted. However, when using 100 μl and 200 μl pipette, whose maximum aspiration pressures are above 3000 Pa, the water under LRs was removed simultaneously with the water under HR, resulting in patterning failure. The results show that aspiration pressures (~ 1950 Pa) slightly greater than ∆*P*_*L*_ under the LR also allow for successful patterning. We expect that further studies of meniscus kinetics using pressure-controlled pumps may explain how successful patterning can still result from aspiration pressures greater than the capillary pressure at interfaces under LRs.

### Throughput and uniformity of the patterning method

The unique nature of this hydrogel patterning method is advantageous in filling multiple devices. To fill a series of devices using conventional pipetting methods, the operator must repeatedly uptake the exact volume of a single device from the stock container before loading the next device. In contrast, the present technique can uptake the combined volume of all desired devices at the onset of loading. Each device is initially overfilled by arbitrarily depressing the pipette prior to aspiration of the excess fluid by releasing the pipette piston. Video S1 shows a comparison of the two patterning procedures to fill a dozen devices that were used for 3D cytotoxicity assay in our previous study^26^. The reduced uptake procedure can enhance experimental throughput when loading hydrogel precursors in microfluidic devices. This is especially beneficial when handling gels with a short pot life, such as fibrin gels whose cross-linking time is approximately 1 min^16^. Using aspiration-mediated patterning, it took around 20 s to fill a dozen devices, while the conventional method that injects the exact amount of hydrogel solution for each device took approximately two-fold longer.

In this manner, the aspiration-mediated patterning method achieves improvements in throughput using a manual micropipette that typically necessitate costlier electronic pipette equipment with automated dispensing capabilities. Furthermore, our experiments show that such electronic pipettes become less reliable with certain chip designs that require small fluid volumes (~ 1 µL) and produce high capillary action. Video S2 provides a side-by-side comparison of patterning eight devices identical to those in Video S1 using three methods: automated injection with an electronic micropipette, manual injection with a standard micropipette, and aspiration-mediated patterning with a standard micropipette. The video provides a bottom view through the transparent PSA film to better visualize the patterning of fluid in the chips. Even though the electronic micropipette in Video S2 was programmed to dispense a uniform volume (1 µL) into each device, the micropipette exhibits a pattern of overfilling one device, underfilling the next, and then again overfilling the subsequent device. We believe that this is due to the high capillary action of the plasma treated microscale structures wicking excess fluid from the pipette into the device. Visual inspection of the micropipette tip after loading each overfilled device consistently revealed an air gap at the tip. Attempting to fill the next well results in ejection of the air into the channel rather than liquid. This ejection of the air primes the micropipette for overfilling of the subsequent device by excess wicking. On the other hand, both manual pipetteing methods successfully pattern all devices, although the aspiration-mediated method provides superior speed.

To further characterize patterning uniformity, we conducted aspiration-mediated patterning with dyed water using a basic 3D-printed device, whose *D*, *h*, and *H* are 600 μm, 100 μl, and 300 μm, respectively. We measured the area of dyed water that remained after aspiration and compared it with the area of 1.2 μl of dyed water injected through one of two ports in the HR. Even though water is injected through a port, the water fills the area under LRs instead of the HR due to large capillary force. Consequently, both methods result in similar positioning of dyed water. The uniformities of the remained dyed water were 92.5% and 89.0% for the aspiration method and manual injection through a port, respectively, and they showed no statistical difference as shown in Figure S2.

### Formation of multiple discrete microchannels via single aspiration

**Figure 3:**
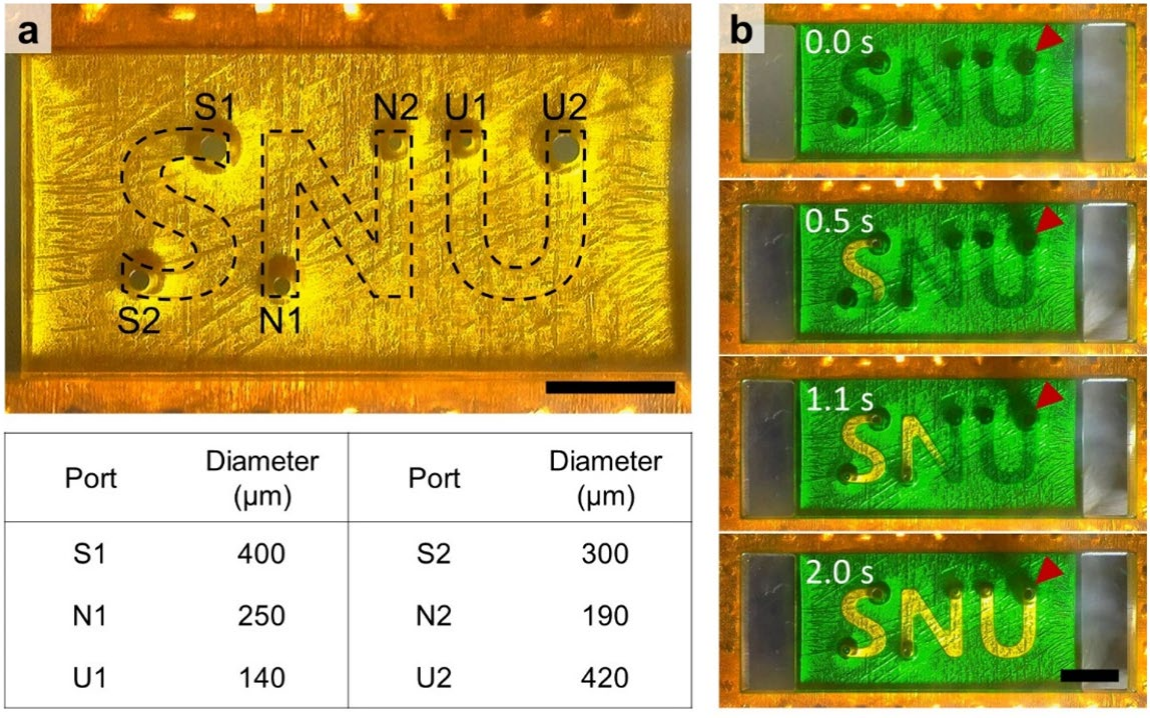
Rendering of multiple discrete microchannels using different port sizes. (**a**) Image of the underside of a device with three HRs in the shapes of ‘S’, ‘N’, and ‘U’, surrounded by a rectangular LR. Loading and aspiration occur through port U2. Opposing port sizes decrease from S1 to U1 as indicated in the bottom table. Scale bar, 2 mm. (**b**) Sequential snapshots (top to bottom) viewed from the bottom of the device during aspiration. Air–liquid interfaces preferentially advance from the largest port to the smallest port. Red arrows indicate the aspirating port, U2. Scale bar, 2 mm.

Our fluid dynamic studies using basic rail structures in the previous sections verify that the air–liquid interface with the smallest critical capillary pressure tends to move first along the rail. Leveraging this phenomenon can provide control over the sequential formation of discrete microchannels with a single aspiration. Figure 3 exemplifies multiple channel formation using various critical capillary pressures at air–liquid interface pinned at holes. In this device, a rectangular LR, 75 μm apart from the underlying PSA film surrounds ‘S’, ‘N’, and ‘U’ shaped HRs, 300 μm apart from the PSA film. The diameter of fluid ports decreases in the order of S1 to U1 as listed in the table in Fig. 3a while U2 has the largest port diameter. When aspirating dyed water filling under the rail structure from the port labeled ‘U2’, the air–liquid interface at the largest opposing port (S1) begins traveling along the rail to form an ‘S’ shaped microchannel (at 0.5 s in Fig. 3b). As the suction continues at U2, the air–liquid interface at the next largest port (N1) now begins to recede (at 1.1 s in Fig. 3b). Consequently, microchannels with shapes of ‘S’, ‘N’, and ‘U’ form via a single aspiration process through one port as shown in Fig. 3b within 2 s (see also Video S3). These results are achievable because the body of fluid underneath the chip is continuous. If instead LRs directly contact the PSA to form solid barriers between HRs, rather than fluid filled gaps, aspiration-mediated rendering of discontinuous microchannels would not be supported.

During multiple channel formation, differences in port diameter within the same channel lead to orderly movement of the air–liquid interface. If ports of the same diameters are employed, air can infiltrate into the channel through the identical ports simultaneously, to form a thin film of liquid between the infiltrating air from the two ports, as shown in Video S4. Breaking the thin film to form a continuous microchannel requires additional pressure drop since foam films have larger Laplace pressures than droplets of equal curvature, owing to the presence of two air–liquid interfaces^30^. In the video, a continuous microchannel forms in the top right HR because it contains the port of aspiration, and thus only a single port for air infiltration. In contrast, a similar device employing asymmetrical port sizes in each HR exhibits unidirectional movement of air–liquid interfaces beneath HRs as shown in Video S5.

**Figure 4:**
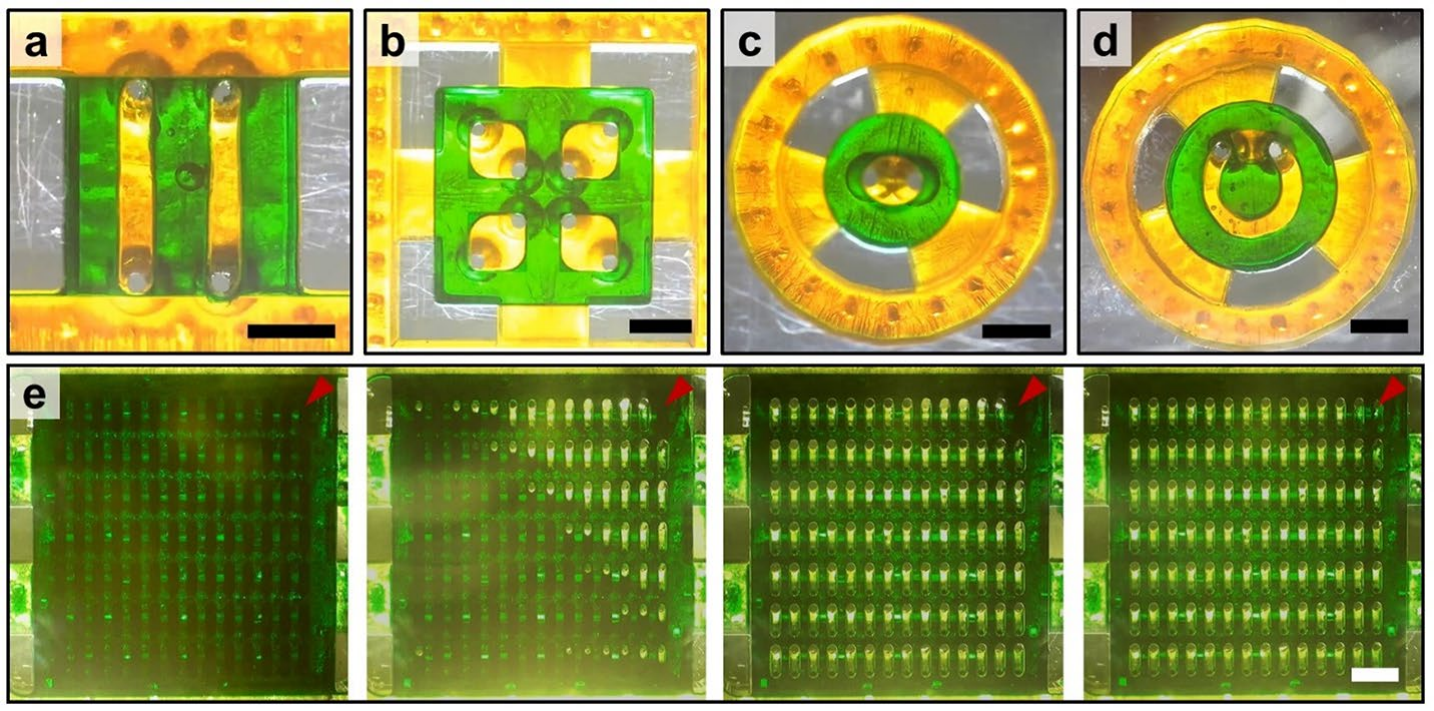
Bottom view images of devices with multiple microchannels with various shapes enclosed by green colored hydrogels. The channels are organized into (**a**) parallel lines, (**b**) a square lattice, (**c**) a circle, and (**d**) concentric circles. (**e**) Time-lapse images from left to right showing the generation of 98 channels via a single aspiration. Since the opposing hole directly beneath the aspirating hole (red arrow) has the smallest hole diameter, hydrogel solution under the channel containing the aspirating hole is removed last. All scale bars are 2 mm.

This unique liquid patterning method enables versatile rendering of microchannel in hydrogels. Figure 4a,b show devices with multiple hydrogel channels arranged in parallel lines and a lattice of squares, respectively. Figure 4c,d respectively highlight the ability of our scheme to generate a single circular and two concentric channels. Figure 4e shows the generation of 98 microchannels under 13.75 mm × 12.7 mm sized low rail via a single aspiration. Video  S6 shows the aspirating process to leave hydrogels under LRs as shown in Fig. 4.

### An open microfluidic device for screening vasculogenic capacities

**Figure 5:**
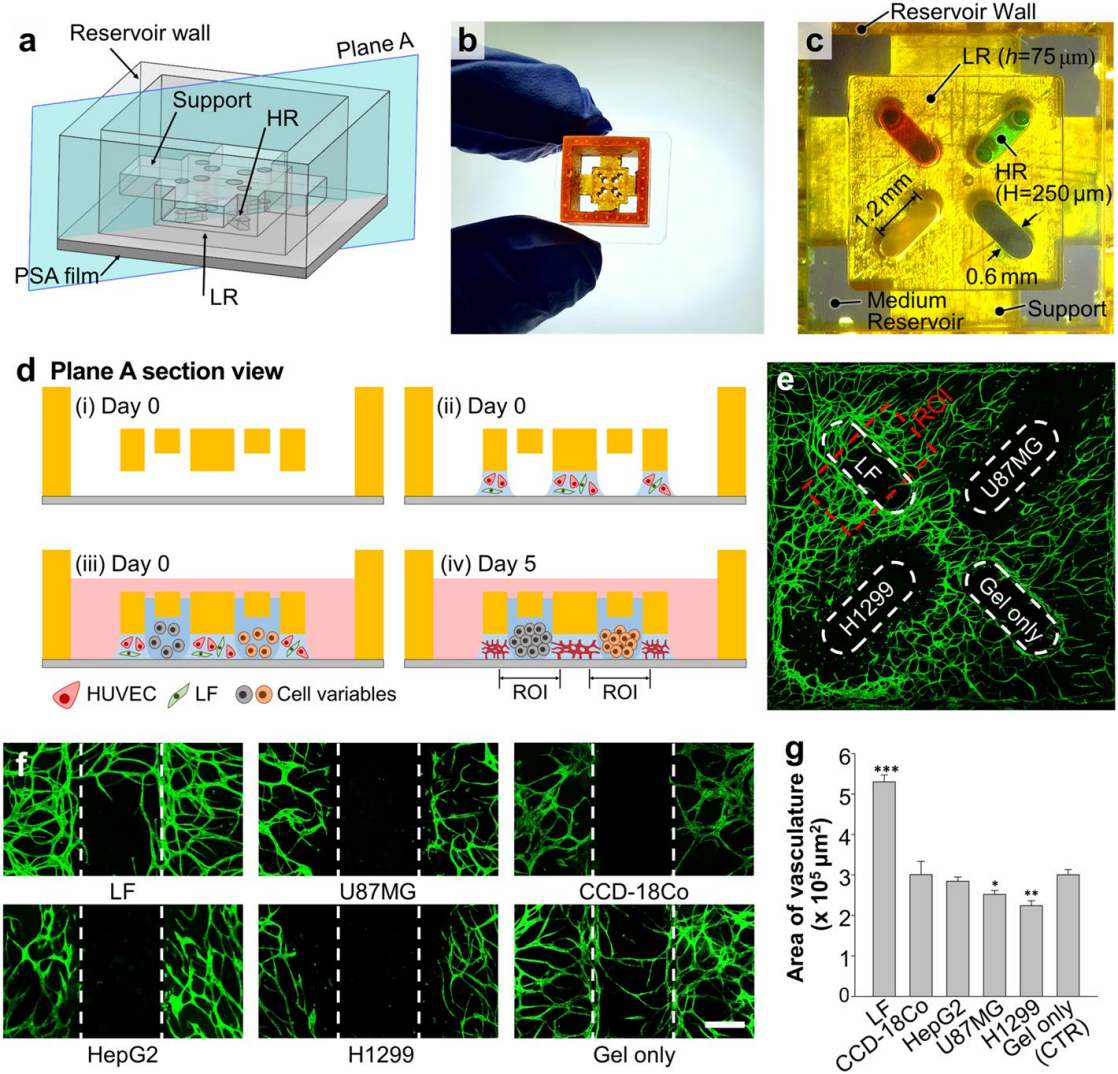
Application of multiple channel rendering for screening vasculogenic capacities of multiple cell types. (**a**) Schematic overview of the device. (**b**) A photo of the device held between two fingers. Edges of a PSA film were not trimmed to highlight that it is bonded to the bottom surface of the 3D printed part. (**c**) Bottom view image of the microfluidic device with four channels surrounded by a square shaped LR (5 mm × 5 mm). (**d**) Schematic diagram of multi-cell co-culture experiment for assessing capabilities to form vascular network. (**e**) Confocal fluorescence image of the vasculogenic co-culture model under the square LR showing CD31 labeled HUVECs with green fluorescence. Red dashed box delineates an example region of interest (ROI) (1 mm × 1.8 mm) centered around the HR, with width equal to three times the HR. (**f**) Representative confocal micrographs of ROIs in channels containing different cell types. Scale bar, 300 μm. (**g**) Plot of average area of vasculature within the ROIs for each cell line obtained from two independent experiments (n = 11 for LF and Gel only, n = 6 for H1299 and U87MG, and n = 5 for CCD-18Co and HepG2). Error bars represent SEM. *** denotes p < 0.001, ** denotes p < 0.01, and * denotes p < 0.05 as measured by one-way ANOVA and comparison against the 'Gel only' condition with Tukey's post-hoc test.

Even though it is widely known that tumors recruit vasculature to acquire oxygen and nutrients^31^, it is not always observed in in vitro models. Previous studies also showed very diverse vasculogenic capacities of cancer cell lines from different organs within an injection-molded device^32,33^, and required additional co-culturing of endothelial cells or fibroblasts to mimic in tumor vasculature^34^. These results guided us to develop a microfluidic model to screen vasculogenic capacities of cell lines for better recapitulation of tumor angiogenesis. Modification of the device in Fig. 4b provides a platform for convenient and effective comparison of paracrine signaling induced by co-culture of five cell types. Here, the microfluidic device contains four linear HRs surrounded by a square LR (Fig. 5a–c). Four support structures suspend the rail structures by connecting them to the reservoir walls, which form growth medium reservoirs when bonded to a PSA film. Patterning of a fibrin hydrogel (2.5 mg/ml) containing a mixture of primary human umbilical vein endothelial cells (HUVEC, 4 × 10^6^ cells/ml) and primary human normal lung fibroblasts (LFs, 1 × 10^6^ cells/ml) under the LR, and subsequent seeding of fibrin gels containing different cell types into the rendered microchannels establishes the basic experimental setup (Fig. 5d). The present studies involve eleven devices, containing microchannels underneath HRs filled with cells from a colon fibroblast cell line (CCD-18Co), a liver cancer cell line (HepG2), a glioblastoma cell line (U87MG), and a lung carcinoma cell line (H1299). In each device, two channels containing either LFs or acellular fibrin gel serve as positive and negative controls, respectively. The results include 11 samples of LFs and control, 6 samples of H1299 and U87MG, and 5 samples of CCD-18Co and HepG2. All gels contain cell concentrations of 5 × 10^6^ cells/ml except for an acellular fibrin gel as a control. Figure 5e presents a confocal image of an exemplary device taken after 5 days of cultivation. Regions of interest (ROI) measuring 1 × 1.8 mm^2^ in area, center around each HR, and encompass the interfaces between the HUVEC-LF gel and the gels containing cells of interest. Analysis of z-projected confocal images yield quantification of the vasculogenic capacities of the cells of interest (Fig. 5f,g). In alignment with previous studies^6,35^, LFs vigorously promote formation of vasculature. Furthermore, acellular gels and gels containing LFs exhibit angiogenic sprouting into the channels. On the other hand, U87MG and H1299 gels inhibit the growth of surrounding vessels and do not provoke cancer angiogenesis. Gels containing CCD-18Co and HepG2 show no significant difference in vasculogenic capacity compared against the acellular matrix. Since tumor angiogenesis is orchestrated by a variety of activators and inhibitors^36^, we hypothesize that the selected cell lines secreted insufficient pro-angiogenic factors in our experimental setup. Even though we could not find a cell line that induces formation of blood vessel networks, LFs significantly showed pro-vasculogenic performance. The results of these tests corroborate the ability of the platform for screening candidates for cancer angiogenesis in vitro. Furthermore, the versatility of the channel rendering method supports facile device adaptation to study a broad range of paracrine signaling cues in various conformations.

## Conclusion

The reported hydrogel patterning method based on interfacial capillary dynamics and associated device design strategies forms the foundation of a new class of aspiration-mediated open microfluidic devices featuring versatile designs and simple operation. The rail structures in these devices, composed of different heights of rails with embedded fluid ports, embody varying critical capillary pressures at each air–liquid interface. Systematic studies corroborated by theoretical models reveal fundamental design rules based on the relationships between geometric parameters of these structures and the capillary pressures. Leveraging these insights serves the development of devices where aspiration selectively removes injected hydrogel precursor beneath high rails to leave behind precisely rendered microchannels. Furthermore, this concept extends to the generation of multiple discrete channels via aspiration from a single fluid port, enabling a wide range of patterning designs with simple fabrication. A demonstrated high-throughput co-culture model for assessing vasculogenic capacities of multiple cell types highlights the practical applications of the multiple channel formation method.

These rail-based microfluidic devices allow simple and robust patterning based on aspiration, dramatically enhancing experimental throughput and mitigating users’ error faced by conventional approaches. When using hydrogel as ECM, simplified patterning process increases the number of samples obtained from a single mixture of hydrogel precursor during cross-linking. The shortened patterning time can also enhance uniformity in properties of hydrogels between samples. Furthermore, the simple geometries of the rail structure are compatible with scalable manufacturing techniques such as injection molding. The enhanced experimental throughput and productivity offered by the outlined strategies can contribute to new high-throughput screening models and accelerate commercialization of microfluidic cell culture models.

## Methods

### Fabrication of open microfluidic devices

Digital light projector (DLP) 3D Printing (perfactory mini 4, EnvisionTEC) produced the bodies of open microfluidic devices with a photo curable resin (PIC100, EnvisionTEC). Rinsing with isopropyl alcohol in an ultrasonic cleaner for 15 min followed by curing ultraviolet curing (1500 W, 2 min) thoroughly solidified the printed pieces. Vapor deposition of parylene (LAVIDA, Femto science) onto the 3D printed parts ensured device biocompatibility. Attaching the bottom side of the printed part to a PSA film completed the open microfluidic devices. Air plasma treatment of the device (70 W, 3 min) prior to fluid injection imparted hydrophilicity to the device surface for facilitating patterning of fluids under rail structures.

### Uniformity test of the patterning method

Three unbiased students performed aspiration-mediated patterning and injection through a hole three times respectively for each method using 3D printed basic rail structures. A digital microscope (AM4115TW, Dino-Lite) captured images after patterning green dyed water under low rails. ImageJ splited the images based on R, G and B values, and wand tool in ImageJ with tolerance value of 60 selected the area of green dyed water in splited images based on G values. We measured the area of green dyed water and calculated mean deviation uniformity following the equation, U = [1 − (Max − Min)/(2Avg)] × 100 (%).

### Cell culture

Cell culture experiments utilized human umbilical vein endothelial cells (HUVEC, Lonza) cultured to passages between 4 and 7 in endothelial growth medium (EGM-2, Lonza), and normal human lung fibroblasts (LF, Lonza) cultured up to passage 7 in fibroblast growth medium (FGM-2, Lonza). CCD-18Co, HepG2, U87MG and H1299 cells were cultured in RPMI 1640 (Thermo Fisher) supplemented with 10% of FBS, penicillin (100 U/ml) and streptomycin (100 U/ml). 0.25% trypsin–EDTA (HyClone) facilitated detachment of cultured cells from the culture dishes. Re-suspension with appropriate amounts of culture medium provided target cell densities before mixing the cell suspension with bovine fibrinogen solutions in ratios of 3:1.

### Hydrogel micropatterning using rail-based microfluidics

3D printed rail structures consisted of high rails surrounded by low rails. HRs housed holes that served as ports for injection of fluids and air infiltration. Injection of fluid via a micropipette through a port in the HR and immediate aspiration generated micropatterned fluid under LRs. Subsequent cross-linking of the solution resulted in patterned hydrogels and adjacent microchannels. Visualization of the patterns employed a solution of bovine fibrinogen (5 mg/ml, Sigma) dissolved in water and mixed with green food dye. Mixing with 50 U/ml of thrombin (Sigma) solution in a 30:1 ratio (v/v) at room temperature began the polymerization reaction of fibrinogen to fibrin. Exposing the loaded device to ambient conditions for 5 min resulted in fully crosslinked green dyed fibrin gel.

For microfluidic co-cultures, fibrinogen solution (10 mg/ml) mixed with cell suspension in ratios of 1:3 (v/v) yielded cellular gels with a final fibrinogen concentration of 2.5 mg/ml. Mixing with thrombin solution (25 U/ml) in a 50:1 ratio immediately before injection into the fluid ports began the polymerization reaction. Immediate patterning and incubation in a cell incubator (37 °C, 5% of CO_2_, 3 min) resulted in fully crosslinked fibrin gel encapsulating cells. Culture of the cellular gels involved adding EGM-2 into each well of the device, followed by incubation for five days with media changes every two days.

### Immunostaining

AlexaFluor^®^488-conjugated mouse monoclonal antibody specific for human CD31 (303110, BioLegend) was used for visualizing blood vessel networks. Cells were fixed with 4% (w/v) paraformaldehyde in PBS for 15 min, and stained with the fluorescence labed antibody diluted to 1:200 in 3% bovine serum albumin for overnight at 4 °C.

### Image analysis

A digital microscope (AM4115TW, Dino-Lite) captured time-lapse images of microchannel formation and images of various microchannel designs. Image analysis using ImageJ of 3D printed bodies provided measured diameters of fluid ports. Confocal microscopy (A1, Nikon) imaged labeled blood vessel networks through optical z-sectioning (100 and 5 μm in depth and interval, respectively). Image analysis for vascularized area measurement utilized Z-projection and conversion to binary images using auto threshold in Fiji following the “Li” method.

### Statistical analysis

One-way ANOVA with pair-wise comparisons by the Tukey post hoc test was used to determine whether six data-sets were statistically significant. At least five samples for each condition within two independent experiments were used for the imaging and data analysis. *** denotes p < 0.001, ** denotes p < 0.01, and * denotes p < 0.05.

## Supplementary Information

Supplementary Information 1.

Supplementary Video S1.

Supplementary Video S2.

Supplementary Video S3.

Supplementary Video S4.

Supplementary Video S5.

Supplementary Video S6.
