# Supplementary material for: Aspiration-mediated hydrogel micropatterning using rail-based open microfluidic devices for high-throughput 3D cell culture
Source: Sci Rep. 2021 Oct 7;11:19986. doi: 10.1038/s41598-021-99387-6 (PMC8497476; doi:10.1038/s41598-021-99387-6)
Supplement: Supplementary file 1 — Supplementary Information 1. [file 41598_2021_99387_MOESM1_ESM.docx]

Supplementary Material

**Aspiration-mediated hydrogel micropatterning using rail-based open microfluidic devices for high-throughput 3D cell culture**

Dohyun Park^1,5^, Jungseub Lee^1^, Younggyun Lee^1^, Kyungmin Son^1^, Jin Woo Choi^1^, William J Jeang^2^, Hyeri Choi^3^, Yunchan Hwang^4^, Ho-Young Kim^*1^, and Noo Li Jeon^*1,3,5^

^1^ Department of Mechanical Engineering, Seoul National University, Seoul 08826, Republic of Korea.

^2^ Department of Materials Science and Engineering, Northwestern University, Evanston, IL 60208, USA

^3^ Interdisciplinary program for bioengineering, Seoul National University, Seoul 08826, Republic of Korea

^4^ Department of Electrical Engineering and Computer Science, Seoul National University, Seoul 08826, Republic of Korea

^5^ Bio-MAX Institute, Seoul National University, Seoul 08826, Republic of Korea

*** Corresponding authors**: Ho-Young Kim (hyk@snu.ac.kr), Noo Li Jeon (njeon@snu.ac.kr)

**Contents of supplementary information**

**Details of calculating pressures of micro-pipettes** (including Figure S1 and Table S1)

**Figure S1.** Experimental setup for measuring aspirating pressure of micropipettes

**Figure S2.** Comparison of patterning uniformity between aspiration-mediated patterning and normal injection through a hole.

**Table S1.** Flow rates, net pressures and aspiration pressures obtained from experiment.

**Video S1.** Experimental throughput comparison of aspiration-mediated patterning with normal injection

**Video S2.** Experimental comparison of aspiration-mediated patterning with normal injection with a manual pipette and an electronic pipette

**Video S3.** Formation of multiple discrete channels via single aspiration

**Video S4.** Formation of thin liquid film within channels with symmetric holes

**Video S5.** Unidirectional removal of liquid under HRs with asymmetric holes

**Video S6.** Formation of multiple discrete channels with various shapes via single aspiration

**Details of calculating pressures of micro-pipettes**

In chapter "Theoretical analysis of microchannel formation", four micropipettes (Pipetman, Gilson) with maximum volumes of 10, 20, 100, and 200 μl and tips (Unfiltered Univeral Tip, Neptune) were used to determine the effect of aspiration pressure. The aspiration pressures of four pipettes were calculated by measuring the flow rates of water through a microfluidic channel with square cross section of 300 μm in length while aspirating the water with each pipette. The microfluidic channel was fabricated by bonding a 3D printed part with silicone adhesive film on the bottom to form microchannels (Figure S1a). Air plasma treatment (70W, 3 minutes) of the device prior to liquid injection imparted hydrophilicity to the device's surface. Once the device was ready, we filled the channel with green dyed water and released the plunger button as quickly as possible to aspirate it into the pipette. While aspiration pressure is applied, the pressure difference (∆*P*) that causes the recession of air-liquid interface is the net pressure of aspiration pressure (∆*P*_a_) and capillary pressure (∆*P*_c_), ∆*P* = ∆*P*_a_ + ∆*P*_c_ (B-B’ secntion view in Figure S1a). Therefore, net pressure difference and capillary pressure should be obtained to calculate the aspiration pressure.

In order to obtain the net pressure, ∆*P*, we measured the speed of the receding interface. Then ∆*P* can be calculated by flow rate using the Hagen-Poiseuille equation which gives the pressure drop of fluid flowing through the pipe.^1^ For a square section microchannel, the equation is simplified to:

$$Q= \frac{Gl^{4}}{12\mu}-\frac{16Gl^{4}}{\pi^{5}\mu}\sum_{n=1}^{\infty} \frac{1}{{(2n-1)}^{5}}\frac{\cosh(\beta_{n}l)-1}{\sinh(\beta_{n}l)}$$

where *G* = ‒ *dp*/*dx* is the pressure gradient, *l* is the side length of a square cross section, μ is the dynamic viscosity, and β = (2n ‒ 1)π/l.^2^ Experimentally measured flow rates allow us to calculate the pressure difference in the microfluidic channel using ∆*P* = *LG*, where *L* is the length of the fluid filled in a channel. The high-speed camera captured time-lapse images of the interface receding at a distance of 3 mm from the aspiration hole at a frame rate of 3000 FPS, which allows the flow rate and pressure drop to be calculated at that location. (Figure S1b).

The capillary pressure in the microchannel, ∆*P*_c_, acts as a resistance to flow and is governed by Young-Laplace equation ∆*P*_c_ = ‒ γκ, where γ is the gas-liquid interfacial tension and κ is the interface curvature. Because the contact angle of the channel walls reaches zero due to plasma treatment, ∆*P*_c_ corresponds to the pressure difference between the hemispherical meniscus with a maximum curvature of 4/*l*: ∆*P*_c_ = ‒4γ/*l* = -959.6 Pa. As a result, the aspiration pressure ∆*P*_a_ or ∆*P* ‒ ∆*P*_c_ is calculated as Table S1 for each micropipette volume.


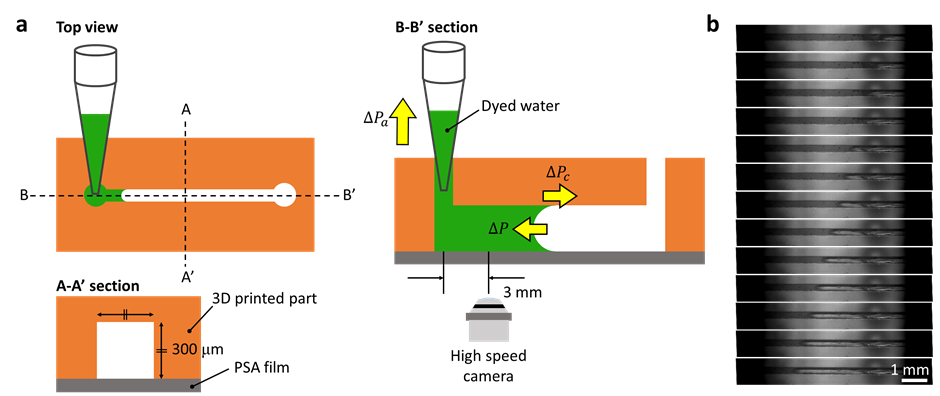


**Figure S1.** Experimental setup for measuring aspirating pressure of micropipettes. (a) A microfluidic device and pressures influencing recession of air-liquid interface. (b) Time-lapse images captured at the point 3 mm apart from the aspirating hole during aspiration with a 10 μl-micropipette with frame rate of 3000 FPS.


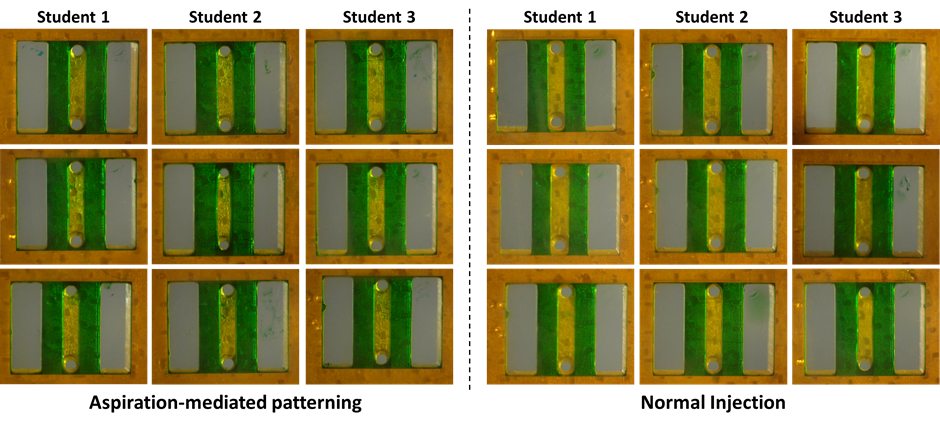


**Figure S2.** Comparison of patterning uniformity between aspiration-mediated patterning and normal injection through a hole. Aspiration-mediated patterning left uniform residues under rail structures regardless of the performer. On the other hand, student 1 loaded smaller amount of dyed water than student 3 via normal injection although they used the same micropipette set to dispense 1.2 μl of water. We guess this difference comes from the water hanging outside the pipette tip which depends on user’s pipetting skill.

| Pipette | 10 ul-pipette | 20 ul-pipette | 100 ul-pipette | 200 ul-pipette |
| --- | --- | --- | --- | --- |
| Q (μl/s) | 93.2 | 98.0 | 268.0 | 421.8 |
| ∆*P* (Pa) | 982.4 | 1032.8 | 2824.5 | 4445.7 |
| ∆*P*_a_ (Pa) | 1942.0 | 1992.4 | 3784.1 | 5405.3 |

**Table S1.** Average flow rates, net pressures and aspiration pressures obtained from experiment (n=3 for each pipette).

**Reference**

1 Sutera, Salvatore P., and Richard Skalak. "The history of Poiseuille's law." *Annual review of fluid mechanics* 25.1 (1993): 1-20.

2 Boussinesq, Joseph. "Mémoire sur l’influence des frottements dans les mouvements réguliers des fluids." *Journal de mathématiques*
